# Longitudinal Auxological recovery in a cohort of children with Hyperinsulinaemic Hypoglycaemia

**DOI:** 10.1186/s13023-020-01438-0

**Published:** 2020-06-24

**Authors:** Chris Worth, Laila Al Hashmi, Daphne Yau, Maria Salomon-Estebanez, Diego Perez Ruiz, Caroline Hall, Elaine O’Shea, Helen Stokes, Peter Foster, Sarah E. Flanagan, Karen E Cosgrove, Mark J Dunne, Indraneel Banerjee

**Affiliations:** 1 Department of Paediatric Endocrinology, Royal Manchester Children’s Hospital, Oxford Road, Manchester, M13 9WL UK; 2 Department of Paediatrics, Nizwa Hospital, Nizwa, Sultanate of Oman; 3 Department of Pediatrics, Division of Endocrinology, Jim Pattison Children’s Hospital, Saskatoon, Canada; 4 Dept of Mathematics, University of Manchester, Manchester, UK; 5 Institute of Biomedical and Clinical Science, University of Exeter Medical School, Exeter, UK; 6 Faculty of Biology, Medicine and Health, University of Manchester, Manchester, UK

**Keywords:** Congenital Hyperinsulinism, Natural history, Height, Weight, Diazoxide, Outcomes, Hypoglycaemia, Neurodevelopment

## Abstract

**Background:**

Hypoglycaemia due to hyperinsulinism (HI) is the commonest cause of severe, recurrent hypoglycaemia in childhood. Cohort outcomes of HI remain to be described and whilst previous follow up studies have focused on neurodevelopmental outcomes, there is no information available on feeding and auxology.

**Aim:**

We aimed to describe HI outcomes for auxology, medications, feeding and neurodevelopmental in a cohort up to age 5 years.

**Method:**

We reviewed medical records for all patients with confirmed HI over a three-year period in a single centre to derive a longitudinal dataset.

**Results:**

Seventy patients were recruited to the study. Mean weight at birth was − 1.0 standard deviation scores (SDS) for age and sex, while mean height at 3 months was − 1.5 SDS. Both weight and height trended to the population median over the follow up period. Feeding difficulties were noted in 17% of patients at 3 months and this reduced to 3% by 5 years. At age 5 years, 11 patients (15%) had neurodevelopmental delay and of these only one was severe. Resolution of disease was predicted by lower maximum early diazoxide dose (*p* = 0.007) and being born SGA (*p* = 0.009).

**Conclusion:**

In a three-year cohort of HI patients followed up for 5 years, in spite of feeding difficulties and carbohydrate loading in early life, auxology parameters are normal in follow up. A lower than expected rate of neurodevelopmental delay could be attributed to prompt early treatment.

## Background

Hyperinsulinism (HI) is a rare group of disorders characterised by dysregulated insulin secretion leading to unpredictable hypoglycaemia. The estimated incidence ranges from 1:2500 to 1:50,000 with higher rates among consanguineous populations [1, 2]. Although rare, HI is the commonest cause of severe, recurrent hypoglycaemia in infancy and childhood [1]. HI is a heterogeneous condition with just under half of patients found to have genetic mutations [3]. In many patients, gene mutations are often not identified, even though genetic aetiology may still be possible. HI can be classified as diffuse or focal, depending on histopathological characteristics following 18F-DOPA positron emission tomography. The distinction of focal HI from diffuse HI is clinically relevant as treatment in the former is predominantly surgical lesionectomy with the possibility of cure. In some patients with HI, reduction in severity of disease is noted [4] with hyperinsulinism being resolving or transient in nature. The cause for transience, even in genetic forms of HI, is not known but is well recognised, particularly in those without identified gene mutations [5].

There are few descriptions of the natural history of HI and most long-term follow up studies have focused on neurodevelopmental outcomes and time to resolution in cross sectional observations only in patients with genetically confirmed HI, ignoring transient forms. The incidence of adverse neurodevelopmental outcomes in patients with HI has been significant - varying between 26 and 44% in large patient groups, and has remained static over the last two decades despite advances in diagnosis and management [4, 6–8]. Poor neurological outcomes have also been found in transient HI patients and do not differ in incidence when compared to patients with persistent HI [9, 10]. Long-term conservative management of HI has been shown to be associated with the remission of clinical symptoms by several groups [5, 8, 11] with the mean time to resolution on diazoxide treatment reported to be 4.8 years [12]. In this subgroup of patients, learning difficulties have been reported in 29% following resolution of symptoms [8].

While considerable data exist to describe relatively large birth weight in neonates with HI [13], there is little natural history data regarding growth after diagnosis. Mazor-Aronovitch et al. described a longitudinal cohort of 21 patients in whom height in follow up was deemed appropriate for parental target heights in 20 patients but did not provide longitudinal auxology data [8]. Similarly, Su et al. describe a “normal growth rate” in a study of 27 patients with HI but without providing specific data [14]. Two studies have described the low risk of growth deceleration in patients receiving octreotide but the focus of these studies was on the medication’s potential for growth suppression rather than the natural history of the disease [12, 15]. Cherian et al. compared weight of post pancreatectomy HI patients with age matched Type 1 diabetes patients and found no significant difference [16]. To our knowledge there is no description in the literature of the long-term height and weight of HI patients from a large cohort.

Therefore, the aims of this study were to review auxology of patients with HI and to review outcomes of medication/disease resolution, feeding and neurodevelopmental status in follow up assessment to describe the longitudinal variation of illness due to HI.

## Methods

All patients treated for HI in our centre over a three-year period between 2011 and 2013 were entered into the NORCHI (Northern Congenital Hyperinsulinism Service) database and followed up over a period of five years. The NORCHI service is a quaternary service for the clinical management of patients with HI in the north of the UK. Patients are referred to NORCHI from regional neonatal units, district general hospitals and other tertiary endocrine centres in the UK.

Patients were included in the study if they had hypoglycaemia secondary to HI, required medical or surgical treatment for HI and had undergone a period of follow up in our centre. The diagnosis of HI was based on the finding of detectable insulin at the time of hypoglycaemia analysed from venous blood samples in association with supraphysiological glucose infusion rates and recurrent episodes of hypoglycaemia without alternative causes, as per criteria described elsewhere [17, 18]. Patients with HI secondary to being born small for gestational age (SGA) were included if HI persisted beyond 2 weeks of life. Patients with HI born to mothers with diabetes/gestational diabetes and those with perinatal stress were excluded. Regardless of birth weight, patients were also excluded if HI resolved within 4 weeks. Gene mutation analysis was performed as per a testing algorithm described elsewhere [19]. Genetic analysis included Sanger sequencing for *ABCC8* and *KCNJ11* as well as targeted gene panel using next generation sequencing. Genetic test results did not form part of inclusion or exclusion criteria as it was felt to be important to capture data from a full cohort of patients with HI.

Data regarding auxology, genetic investigations, neurodevelopmental status and feeding outcomes were obtained by reviewing the database and electronic patient records. Five year follow up data was obtained as close to the fifth birthday as possible. Patients (*n* = 2) who presented outside the neonatal period also had data measured at birthday milestones rather than time since diagnosis.

Following discharge from the hospital, patients were reviewed in the outpatient department where they were always weighed and measured using the same measuring board/stadiometer and weight scales by a variety of trained observers. Additional information was retrospectively obtained from patient records. Longitudinal outcomes included height, weight, medication dosages and method of feeding at 3-, 6-, 12-, 24-, 36-, 48- and 60 months. Neurodevelopmental outcomes were reviewed at 60 months only. Neurodevelopmental problems were classed as none, some or severe as described previously [9] through a combination of formal psychometric testing and neurodevelopmental assessment by a developmental paediatrician. Some neurodevelopmental delay was defined as mild cognitive delay or learning difficulties. Severe neurodevelopmental problems were classed as seizures, autism, cortical blindness, cerebral palsy or severe developmental delay. Feeding problems were classified as requirement for nasogastric (NG) or gastrostomy tube to deliver at least a proportion of feeds every day. Auxology data were converted to standard deviation scores (SDS) using population specific data for UK children [20]. Disease was classed as persistent if the patient had ever undergone surgery or was requiring medication to treat hypoglycaemia at that time point. Transient disease was classed as spontaneous resolution of disease. In the absence of a uniform and/or consensus definition of transient HI and the recognition that resolution of HI can be both early and late [4, 5], transience of HI was not time limited but assessed within the longitudinal model of the study. Resolution was defined as having undergone an age appropriate fast off medication with no hypoglycaemia.

The study design was observational with no a priori hypothesis in the absence of previous comparable data and/or longitudinal observations in HI cohorts. Power calculations were not performed for these reasons.

### Statistical analysis

**Fig. 1:**
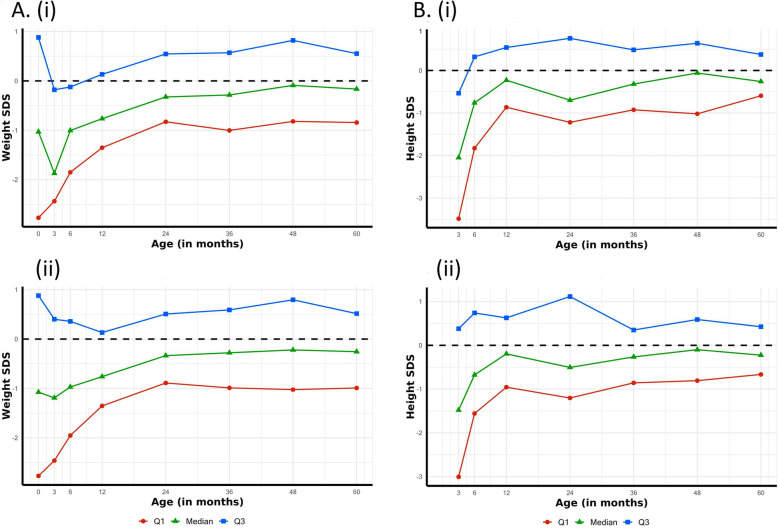
Graphical representation of median and interquartile range for weight and height values from raw and imputed data. The graphs above demonstrate the reliability of linear interpolation used for data imputation in this study. As can be seen, there are no changes in the trends for weight (**a**) and height (**b**) when imputed data (i) were used compared with raw data (ii) alone

Patients who had ≥5 missing weight values or ≥ 4 missing height values (more than half of available observations) were excluded from analysis for that outcome. For patients with < 5 or < 4 missing values for weight and height respectively, imputation was used to complete data sets. Seven separate simulations were run having systematically excluded 1, 2, 3 values for height and 1, 2, 3, 4 values for weight and then five different imputation methods were used to predict the missing values: Splines, Mean, Median, Linear and Last Observation Carried Forward. Complete cases were then used to validate the imputed value against the true value. Accuracy of imputation was measured using mean square error, mean absolute error, root mean square error and mean absolute percentage error. After 100 iterations had been run, linear interpolation was found to be the most accurate. This was then used to calculate missing variables for weight and height data for those patients included in analysis (Fig. 1).

Remaining data were analysed by IBM SPSS 25. Mann-Whitney U tests were used to assess for differences in average insulin levels and early maximum diazoxide dose between subgroups. Chi-square and Fisher’s Exact tests were used to assess for differences in percentages of patients with permanence of HI between subgroups such as high or low maximum early diazoxide dose.

The study was conducted as per local research ethical approval (reference 07/H1010/88).

## Results

### Genetic profiling

**Fig. 2:**
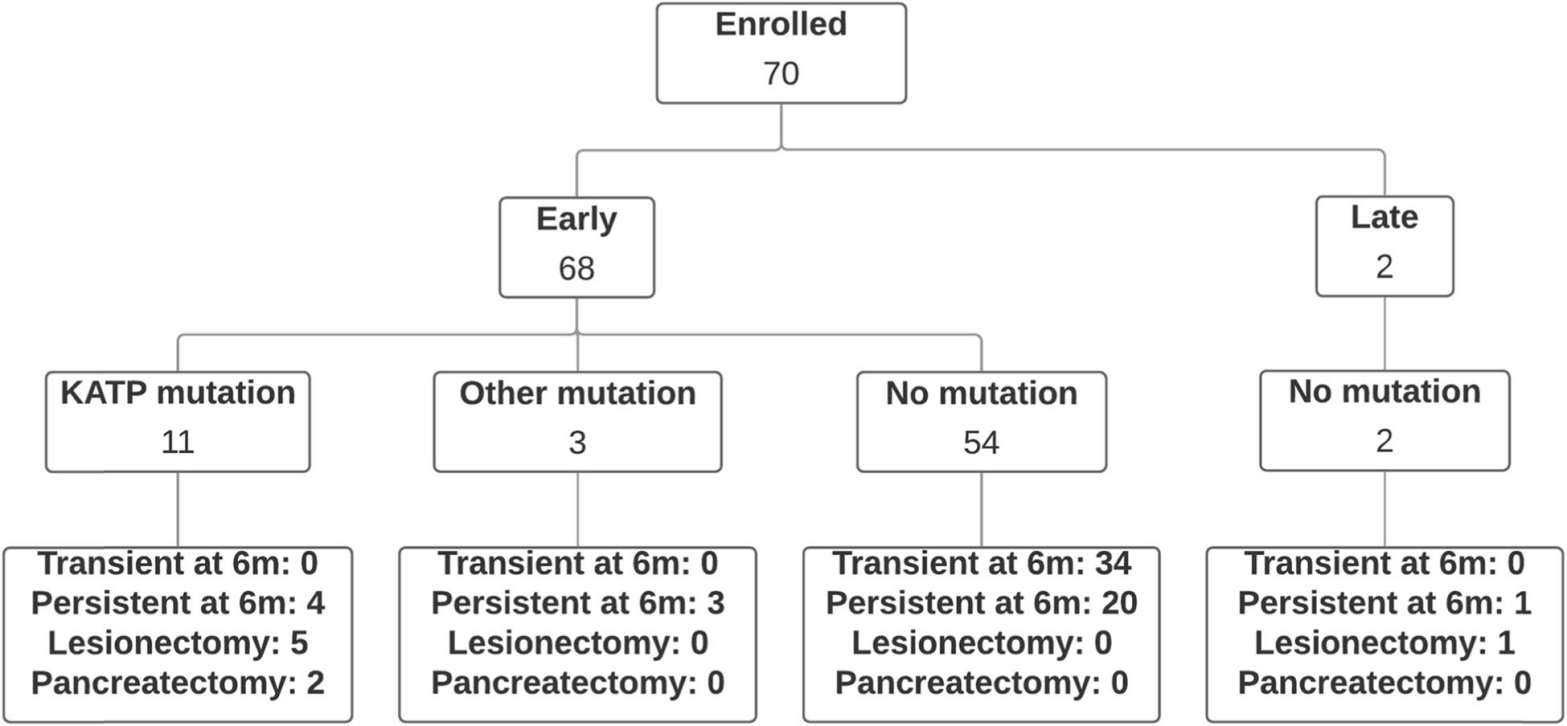
Flowchart of natural history outcomes of HI. Frequency of patients in each category determined by time of presentation, mutation status, transient/persistent and surgical outcomes

Seventy patients were enrolled into the cohort study (Fig. 2). Only two patients were diagnosed outside the neonatal period and were aged 8 months and 14 months at diagnosis. HI was diagnosed with mean (interquartile range (IQR)) insulin level 73 (67) pmol/L at hypoglycaemia with a mean (IQR) plasma glucose of 1.6 (1.2) mmol/L. In fourteen (20%) patients a disease-causing variant was identified by Sanger sequencing or targeted next generation sequencing; genetic testing was not performed if patients demonstrated a resolving course of disease. Eleven patients had disease-causing variants in the *ABCC8 or KCNJ11* gene*,* which encode the pancreatic K-ATP channel, while the rest had mutations in *HNF4A* (two patients) and *GCK* (one patient). Six patients required a lesionectomy for the treatment of focal HI and two required subtotal pancreatectomy for non-focal HI.

### Auxology profiles

**Fig. 3:**
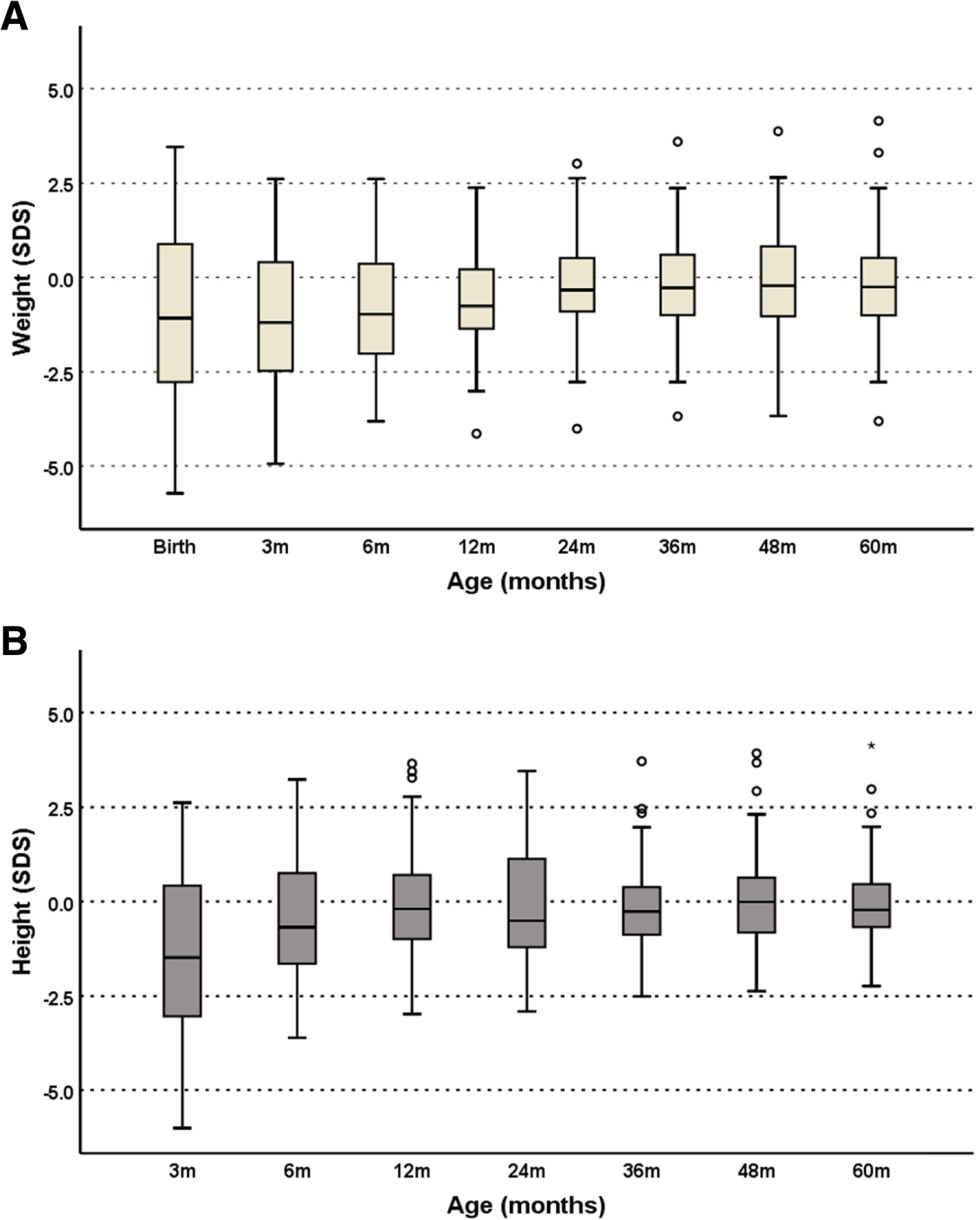
**a** Box and whisker plot analysis of median weight SDS by age. This demonstrates a lower than average birth weight with a trend towards the population median weight at 24 months. Each box extends from the lower quartile to the upper quartile of the data used with the horizontal line in the box indicating the sample median. The whiskers extend to the smallest and largest observations within 1.5*IQR from the lower and upper quartiles, respectively. Any data points plotted beyond the ends of either whisker (as circles) are regarded as possible outliers. **b**. Box and whisker plot analysis of median height SDS by age. This demonstrates an initially lower than average height, in keeping with weight, with a trend back to population median height. Each box extends from the lower quartile to the upper quartile of the data used with the horizontal line in the box indicating the sample median. The whiskers extend to the smallest and largest observations within 1.5*IQR from the lower and upper quartiles, respectively. Any data points plotted beyond the ends of either whisker (as circles) are regarded as possible outliers

Full set weight data (measured value available for every time point) was obtained for 32 patients while linear interpolation was used to impute missing values for another 28 (Fig. 3a). Ten patients were excluded from longitudinal analysis due to insufficient available measurements (missing more than half their possible measurements). Mean (IQR) weight SDS at birth was − 1.00 (3.65) with reduction at age 3 months to − 1.27 (2.89) followed by recovery to − 0.06 (1.87) by age 4 years when it remained stable until age 5 years (Fig. 3a). Of the cohort, only four patients (7%) had weight SDS > + 2.0 at 5 years. Mean weight SDS at birth was − 3.2 for SGA patients and + 0.4 for non-SGA patients. For non-SGA patients, birthweight SDS was similar in those with and without genetic mutations (*p* = 0.098 for difference between the two).

Full set height data was obtained for 30 patients while linear interpolation was used to impute missing values for another 22 (Fig. 3b). Eighteen patients were excluded in longitudinal analysis due to insufficient available measurements (missing more than half their possible measurements). Mean (IQR) height SDS at 3 months was − 1.5 (3.5) which increased to − 0.06 (1.8) at 12 months and remained stable until 5 years with a height SDS of + 0.05 (1.2) (Fig. 3b). Figure 3a and b show cross sectional summary measurements at each follow up to demonstrate auxology increments in review appointments.

Only five patients received octreotide after discharge from hospital and all achieved a normal height by the age of 5 years (range − 0.3 to + 1.3 SDS for height). There was no significant difference between height SDS during and after treatment with octreotide.

**Fig. 4:**
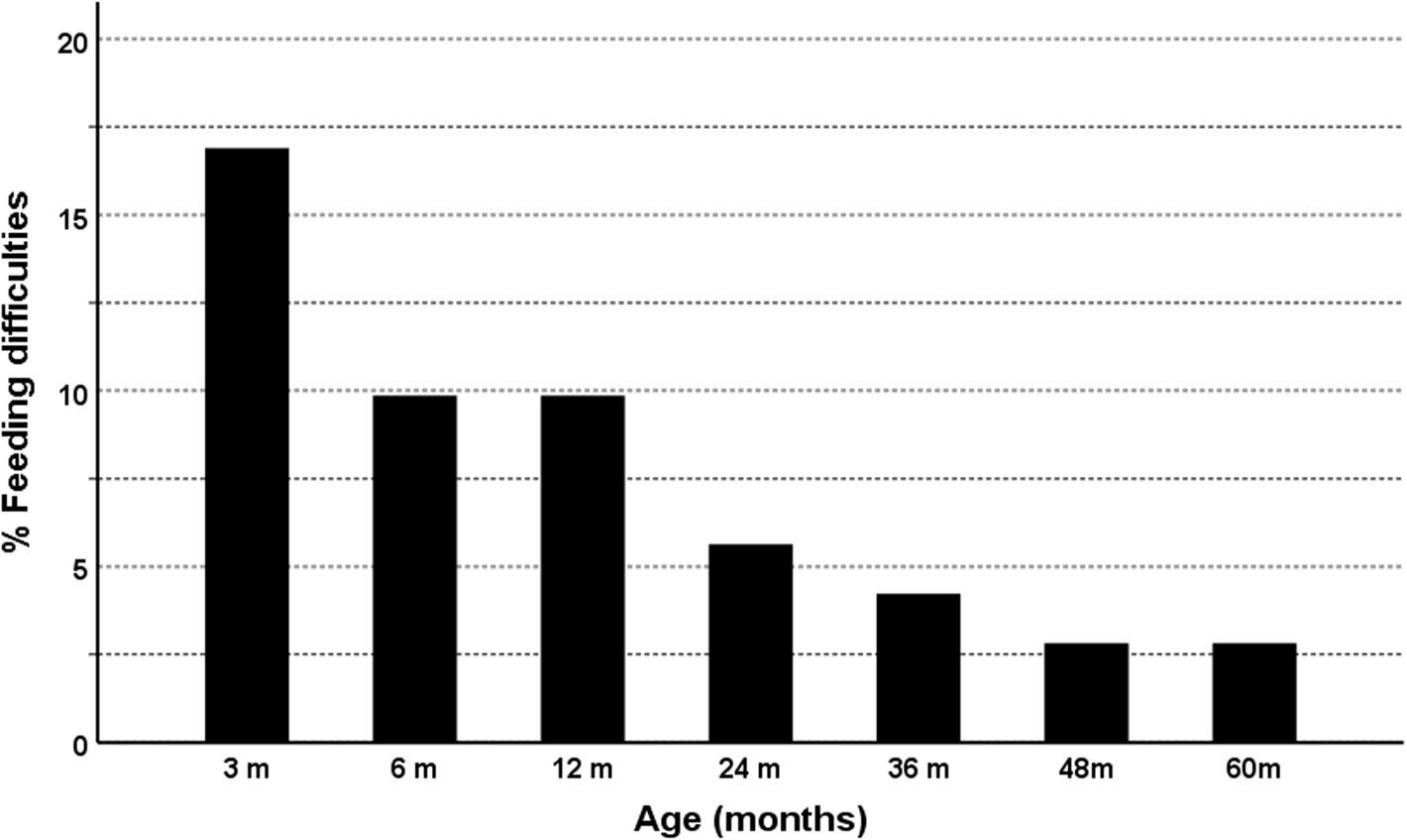
Percentage of children with feeding difficulties by age. Demonstration that the percentage of children with HI and feeding difficulties reduced from a maximum of 17% at 3 months of age to only 3% at 48 months of age. Feeding difficulties were classified as requirement for nasogastric or gastrostomy feeding

There were 12 children (17%) with feeding problems at 3 months and a steady reduction was observed over time with only two children (3%) having problems with feeding by 4 years of age, a figure which persisted until 5 years (Fig. 4). The two patients with persistence to 5 years both had diffuse HI. One of these patients had a dominantly-acting de novo *ABCC8* mutation.

### Neurodevelopmental outcomes

At age 5 years, neurodevelopmental delay was documented in 11 patients (15%); these were either mild cognitive delay or learning difficulties at school (defined as some neurodevelopmental delay) in ten patients. However, one patient was found to have severe neurodevelopmental delay and also developed autism during the follow up period. One of the patients with neurodevelopmental delay had a paternally inherited *ABCC8* mutation and focal CHI, all others had no identified mutation and non-focal CHI. There was no statistically significant association between neurodevelopmental outcomes and either the presence of a genetic mutation or focal CHI.

### Diazoxide as a severity and prognostic marker

All patients were started on diazoxide as a first line treatment for HI. The mean (IQR) initial maximum diazoxide dose (mg/kg/day) was significantly higher in focal versus non-focal HI (11.8(6.3) vs 6.4(2.4), *p* = 0.003) as well as in those with a K_ATP_ mutation versus those without (11.1(8.8) vs 5.9(1.5), *p* < 0.001) and in those who required surgery versus those who did not (11.3(8.8) vs 6.3(8.8), *p* = 0.001). Mean (IQR) initial maximum dose (mg/kg/day) was also higher in those with persistent versus transient HI at 6 months (8.0(5.8) vs 5.6(0.7), *p* = 0.007) and at 5 years (9.4(10.0) vs 6.4(2.5), *p* = 0.015), indicating the high value of diazoxide dosage as a severity and prognostic marker in the early stages of diagnosis. A cut off maximum diazoxide dose of 5 mg/kg/day was found to be a predictor of spontaneous resolution at 6 months and at 5 years [Table 1]. Patients receiving > 5 mg/kg/day of diazoxide soon after diagnosis to stabilise hypoglycaemia had a higher probability of persistent disease at 6 months (62% vs 32%, chi square = 5.895, p = 0.015) and at 5 years (27% vs 7%, chi square 5.395, *p* = 0.02) than those receiving ≤5 mg/kg/day.

**Table 1:** Resolution of HI in relation to maximal diazoxide dose

Resolution of HI	Diazoxide < 5 mg/kg/day (*n* = 44)	Diazoxide > 5 mg/kg/day (*n* = 26)	*p*-value for difference
Resolution at 6 months n (%)	30 (68%)	10 (38%)	0.015
Resolution at 5 years n (%)	41 (93%)	19 (73%)	0.020

A diazoxide dose < 5 mg/kg/day was associated with a greater tendency to resolution both at 6 months and at 5 years. Significant differences are shown as *p* values for Chi-squared tests

### Resolution of HI

**Fig. 5:**
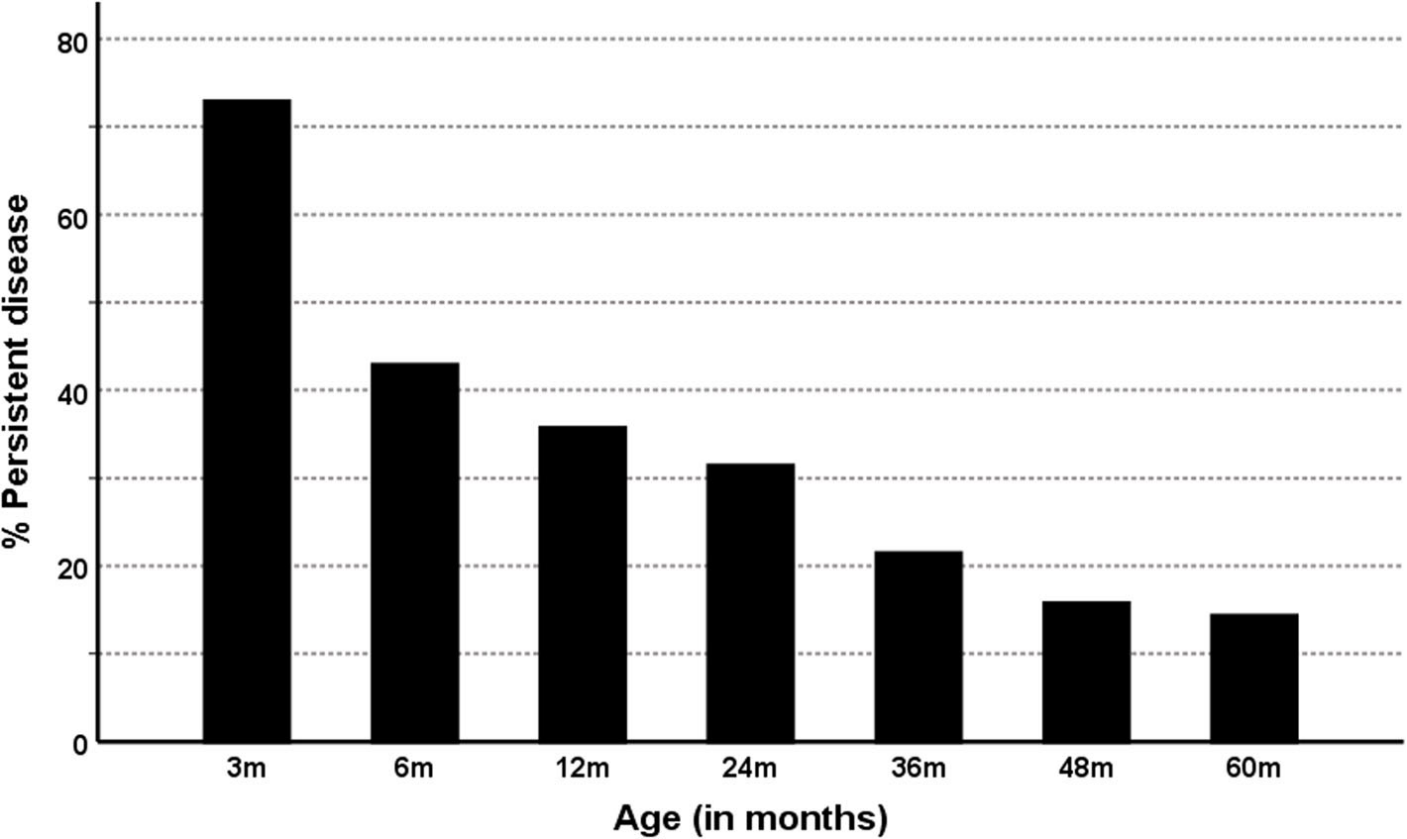
Percentage of children with persistent disease. Children either requiring medication or who had undergone surgery were classed as having persistent disease. This demonstrates the steady improvement in spontaneous resolution of patients with HI

Spontaneous resolution was observed in 19 patients (27%) at 3 months of age. This number steadily rose to 60 patients (86%) with spontaneous resolution at 5 years of age (Fig. 5). A further eight patients underwent surgery and of these, seven were able to stop all medication by age 5 years. This left only three patients (4%) requiring medication for hypoglycaemia aged 5 years, one of whom had undergone a subtotal pancreatectomy.

Babies with HI who were born small for gestational age (SGA) had a lower chance of persistence of disease at 6 months than those not born SGA (29% vs 38%, *p* = 0.009). This continued to 5 years of age where SGA children had a much lower chance of persistence of disease than those not born SGA (0% vs 23%, *p* = 0.012).

## Discussion

We have described auxology, medication, feeding and neurodevelopmental outcomes in a 5 year longitudinal cohort of patients with HI in a single centre. While neurodevelopmental outcomes and time to resolution have been reported in many studies, there are no reports of the natural history of HI with regards to height, weight, feeding or medication.

The median birth weight of this cohort was below average, contrary to typical descriptions of patients with HI being large at birth. The reasons for lower than expected birth weight is not clear but could be due to the increased frequency of HI secondary to SGA birth weight babies. However, in subset analysis, patients not born SGA were also not large at birth, with a mean weight SDS of + 0.4. In the context of a relatively low birth weight and liberal use of carbohydrates to prevent and treat hypoglycaemia, we did not demonstrate early life excess weight gain tracking through to later childhood. Height was very well preserved in our patients indicating that nutritional management of hypoglycaemia does not adversely impact upon growth. An initially low SDS for height at three months recovered quickly and patients achieved a normal height at 5 years. Although we have demonstrated satisfactory auxology in a 5 year follow up, the longer-term outcomes remain unknown. Longer follow up studies in diverse populations will be required to address risk in adult life.

Feeding problems are frequent in many patients with HI in early life [21] and these may persist for many years [22]. The requirement for supra-physiological calorie intake (via artificial enteral and intravenous dextrose administration [23]), diazoxide induced-nausea and reduced opportunities for breast or bottle feeds creates an environment primed for infant feeding problems [24]. Despite input by our Multidisciplinary Team (MDT) comprising dietetic and speech and language therapy, 17% of patients with HI required some form of artificial feeding at 3 months. This frequency reduced over time with continued MDT input; by 4 years of age 97% were feeding orally without supplementation. This data is important in determining the risk of long-term harm from early life treatment as, at an age of developmental plasticity, modification of energy and carbohydrate intake may have profound implications for the risk of obesity and cardiovascular disease in later life [25].

The prevention of neurodevelopmental problems secondary to hyperinsulinaemic hypoglycaemia is a cornerstone of HI management. In spite of treatment advances, there has been only a minimal reduction in the rate of this complication over the last two decades^5,6^. In our cohort, we demonstrated a lower rate of neurodevelopmental problems (15%), most of which were mild, than in other studies^7^. The cause for a reduction in neurodevelopmental problems has not been explored in this study but could be a consequence of the impact of early detection and prompt treatment of hypoglycaemia promoted at our centre. It is possible that our relatively high rate of transient HI may account for this reduction. However, previous studies have shown no difference in neurodevelopmental outcomes between those with transient and permanent HI [9].

In the absence of a marker of disease severity, predicting long-term outcomes for the many subgroups of patients with HI is challenging. In this study, we found that the maximal diazoxide dose around the time of diagnosis had a real prognostic value. A higher dose correlated with a greater likelihood of disease persistence, indicating that dose may be used as a proxy marker along with SGA status to determine long-term outcome. In a disease for which it is notoriously difficult to predict outcomes this information will help in counselling families and planning treatment. As there is no standard marker of a “good response” to diazoxide, having a cut off value is also helpful for future prediction and can be easily explained to parents.

Another factor with predictive value on long term resolution or persistence was whether the patient had been born SGA. Recent studies have suggested the presence of SGA as a predictor of earlier resolution [26] and our data are entirely consistent with this. However, not all patients born SGA underwent resolution of HI within a few weeks; 29% of SGA patients continued to have HI beyond 6 months. The cause for this observation has not been explored in our study but SGA status used with early maximum diazoxide dose could help plan treatment options and give families some realistic projections of expected outcomes.

We recognise that a potential limitation of our study is the relatively high numbers of patients with transient HI and without confirmed genetic mutations. However, our study reflects the real-world nature of cohorts of patients with HI unaffected by referral bias skewed towards the most severe and persistent of patients. Further, inclusion of patients with SGA and Transient HI has several advantages. Inclusion enables an unselected and unbiased review of frequency of disease persistence in longitudinal follow up and provides valuable insight into the effect of supraphysiological nutritional supplementation and growth restricting therapies at a critical plastic period in human development. We acknowledge that our study criteria exclude babies born SGA in whom HI resolved within 2 weeks; therefore, our study inferences regarding auxology, medication, resolution and feeding are not generalisable to all SGA babies.

## Conclusions

We have detailed the first description of a five-year longitudinal cohort of HI patients to show maintenance of auxological parameters without risk of early life obesity secondary to excess carbohydrate usage in early life. Height is well preserved amongst this group implying provision of adequate nutrition for growth. Feeding problems occurred in a significant proportion but the frequency reduced with sustained multidisciplinary interventions. The incidence of patients with neurodevelopmental delay at 5 years was lower than previously reported, implying an optimistic shift from previous high rates over the past decade.

## Data Availability

The datasets used and/or analysed during the current study are available from the corresponding author on reasonable request.
